# Eco-friendly spectrofluorimetric determination of remdesivir in the presence of its metabolite in human plasma for therapeutic monitoring in COVID-19 patients

**DOI:** 10.1038/s41598-025-05198-4

**Published:** 2025-06-23

**Authors:** Mai H. Abd El-Fattah, Yasmine Ahmed Sharaf, Heba M. El-Sayed, Said A. Hassan

**Affiliations:** 1 Pharmaceutical Analytical Chemistry Department, College of Pharmaceutical Sciences and Drug Manufacturing, Misr University for Science & Technology, 6th of October City, Giza, 12566 Egypt; 2 Department of Analytical Chemistry, Faculty of Pharmacy, Zagazig University, Zagazig, 44519 Egypt; 3 Department of Pharmaceutical Analytical Chemistry, Faculty of Pharmacy, Cairo University, Cairo, 11562 Egypt

**Keywords:** COVID-19, Eco-friendly, Remdesivir, Spectrofluorimetry, Stability indicating, Therapeutic drug monitoring, Diseases, Chemistry

## Abstract

The global outbreak of the novel coronavirus (COVID-19) has highlighted the urgent need for innovative therapeutic solutions. Remdesivir (REM) was the first drug granted approval by the US FDA for treating hospitalized COVID-19 patients. A selective and sensitive derivative spectrofluorimetric method has been developed and validated for the determination of Remdesivir (REM) in presence of its Alkaline-induced degradation product (AKDP), which is also known to be its metabolite (GS-441524). The method utilized the intrinsic fluorescence properties of REM, achieving a linear response within the range of 3.0–120.0 ng/mL at 428.3 nm using first-order derivative. Methodological parameters were optimized to ensure high sensitivity, with detection and quantification limits of 1.12 and 3.67 ng/mL, respectively. This approach successfully quantified REM in pure form, intravenous infusions, and spiked human plasma. Recovery rates in plasma were satisfactory at 97.64 ± 1.87, confirming the method’s suitability for therapeutic drug monitoring (TDM) in COVID-19 patients. Additionally, the environmental sustainability of the method was evaluated using GAPI, AGREE, and RGB12 metrics, underscoring its green and eco-friendly characteristics.

## Introduction

In the twenty-first century, the COVID-19 pandemic was triggered by the Severe Acute Respiratory Syndrome Coronavirus 2 (SARS-CoV-2), a single-stranded RNA virus with a rapid transmission rate and a high infection rate. This has led to widespread global infections and elevated mortality rates^1^. SARS-CoV-2 represents the third coronavirus in recent decades to infect humans, following the outbreaks of SARS and Middle East Respiratory Syndrome (MERS). During the outbreak, numerous treatment approaches were employed, including several drug classes, and convalescent plasma therapy, often in combination with invasive and non-invasive oxygen support^1^. Due to the lack of specific antivirals for COVID-19, researchers have turned to drug repurposing, utilizing previously approved antiviral agents to combat the rapid spread of the virus^2^.

**Fig. 1:**
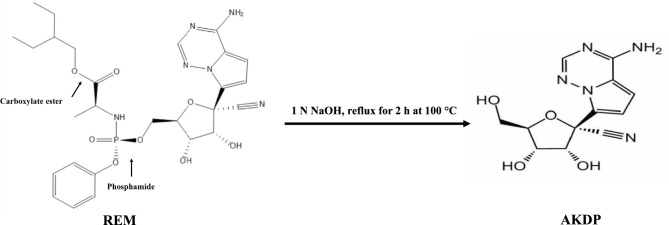
Suggested degradation pathway of Remdesivir.

Remdesivir (REM), chemically identified as 2-Ethylbutyl (2S)-2-[[(S)-[[(2R,3S,4R,5R)-5-(4-aminopyrrolo[2,1-f][1,2,4]triazin-7-yl)-5-cyano-3,4-dihydroxytetrahydrofuran-2-yl] methoxy](phenoxy)phosphoryl]amino]propanoate (Fig. 1)^3^, is a prodrug that is metabolized into its active form, GS-441524, upon administration. GS-441524 inhibits viral RNA-dependent RNA polymerase by competing with ATP for RNA incorporation, thereby blocking RNA transcription and reducing viral RNA replication^4^. This mechanism grants REM broad-spectrum antiviral activity against several RNA viruses, including the Ebola virus, MERS-CoV, SARS-CoV, and SARS-CoV-2^5^. The emergence of SARS-CoV-2 in 2019 renewed interest in REM due to its antiviral properties. It was the first drug to demonstrate efficacy against SARS-CoV-2 in clinical trials, leading to its approval for COVID-19 treatment by the FDA and the European Medicines Agency^6^. REM’s mechanism of action and broad antiviral spectrum make it a pivotal component of antiviral therapy, with ongoing research exploring its potential use in combination therapies^7^.

Due to its near-complete first-pass metabolism, REM exhibits poor oral bioavailability and is thus administered intravenously (IV). It is typically infused in 0.9% saline over 30 to 120 min. The maximum plasma concentrations (C_max_) of REM and its primary metabolite, GS-441524, have been reported in the ranges of 2610–5440 ng/mL and 140–150 ng/mL, respectively^8^. REM is an inhibitor of several cytochrome P450 enzymes, potentially leading to numerous drug interactions when included in treatment regimens^8^. For example, REM may reduce the metabolism of aminophylline and the excretion of acyclovir^4^. Consequently, therapeutic drug monitoring (TDM) is crucial for patients receiving REM, especially those on multiple concurrent medications.

REM is available as either a concentrate or a powder for infusion. While the powder formulation can be stored at temperatures below 30 °C, the concentrate must be stored frozen, thawed, and diluted prior to IV administration. This requirement arises from REM’s susceptibility to hydrolysis. Stability studies have revealed two hydrolysis-related impurities, one of which is the active metabolite^3^. Furthermore, the product undergoes sterile filtration to mitigate degradation, as autoclaving leads to significant deterioration of REM. Moreover, the chemical stability of the diluted infusion is only confirmed for up to 24 h^3^. Therefore, stability-indicating assays for REM in its formulations and diluted solutions are essential during COVID-19 therapy.

Analytical chemists aim to develop highly sensitive and specific methods that specifically target compounds of interest in the presence of their metabolites and/or degradation products. TDM often requires the ability to detect drugs at nanogram levels in biological fluids in presence of potential metabolites. Techniques such as chromatography, spectroscopy, and electrochemistry are commonly employed for stability studies and TDM^9–12^. Despite their effectiveness, chromatographic methods face several challenges, including high costs, bulky equipment, time-consuming processes, and environmental concerns due to solvent use^13–16^. As laboratories move toward sustainability, there is increasing interest in alternatives to chromatography for real-time applications like TDM and degradation monitoring^17–19^.

Spectroscopic methods offer a promising alternative due to their lower operational costs and simpler handling. However, the spectral overlap between drugs and their degradation products or metabolites remains a challenge, often arising from structural similarities. Advanced spectral manipulation techniques can address this issue^20–24^. Fluorescence spectroscopy, in particular, has gained attention for its superior analytical performance and environmental sustainability compared to chromatography. This technique offers higher sensitivity and selectivity, and it facilitates the use of green solvents, making it an ideal choice for stability studies and TDM^25^.

Several methods for REM determination have been reported, including spectrophotometry^26,27^, spectrofluorimetry^28–30^, chromatography^31–35^, and electrochemical techniques^36,37^. However, existing fluorometric methods have not been optimized for detecting REM in the presence of its metabolites or degradation products. Furthermore, some stability studies lack eco-friendly approaches or rely on expensive instrumentation, such as liquid chromatography-mass spectrometry (LC–MS), which is not commonly available in quality control (QC) or hospital laboratories. These limitations underscore the need for a new method capable of determining REM alongside its metabolites or degradation products.

The purpose of this manuscript was to develop a specific, sensitive, and environmentally friendly spectrofluorimetric method for the determination of REM in the presence of its metabolites or degradation products. The proposed method is intended for TDM of REM and monitoring its stability in IV infusions during COVID-19 treatment protocols.

## Experimental

### Instrumentation

All measurements were recorded using a SHIMADZU RF-6000 spectrofluorometer (Tokyo, Japan), operated with Lab Solution RF software version 1.01. The fluorometer featured two monochromators for excitation and emission wavelengths ranging from 200 to 600 nm and utilized a 150 W xenon lamp. A JENWAY pH meter was employed for pH measurements.

### Materials and reagents

All reagents and chemicals used were of analytical grade. Hydrochloric acid and organic solvents, including acetone, methanol, ethanol, n-hexane, and propylene glycol, were procured from PIOCHEM (Egypt). Surfactants such as polyvinyl alcohol and poloxamer 407 were obtained from Sigma-Aldrich (Germany), while sodium hydroxide was supplied by VWR Chemicals (USA).

Remdesivir (100.55 ± 1.21%) was provided by the Rameda Company (Egypt). Additionally, Remdesivir® IV (batch no. 203526), manufactured by the same company and labeled to contain 100 mg/20 mL of REM, was purchased from a local pharmacy.

Human plasma was supplied from the Holding Company for Biological Products and Vaccines, Egypt. No human subjects were involved.

### Solutions

#### Preparation of REM stock and working standard solutions

In a 100-mL volumetric flask, stock standard solution of 100 µg/mL was prepared using distilled water. This stock solution was further diluted to prepare a working standard solution of 100 ng/mL using distilled water.

#### Preparation of Alkaline-induced degradation product (AKDP) stock and working solutions

To prepare AKDP, 25 mg of pure REM was placed in a round-bottom flask with 25 mL of 1 N NaOH. The mixture was refluxed in a water bath at 100 °C for 2 h. After cooling, the solution was neutralized with 1 N HCl and diluted to 100 mL with distilled water in a volumetric flask, yielding an AKDP stock solution equivalent to 250 µg/mL REM. This stock solution was further diluted to prepare a working solution of 100 ng/mL using distilled water.

### Procedure

#### Spectral characteristics

The excitation and emission spectra of REM and AKDP solutions (50 ng/mL each) were recorded against distilled water as a blank over the wavelength range of 200–600 nm. The emission spectra were recorded following excitation at λ_ex_ = 245 nm.

#### Construction of the calibration curve

Aliquots equivalent to 30–1200 ng REM were accurately transferred from their the working standard solution (100 ng/mL) into a series of 10-mL volumetric flasks and completed to volume with distilled water to achieve a final concentration range of 3–120 ng/mL. Fluorescence spectra of the prepared solutions were recorded between 246 and 600 nm and saved. Bandwidth was 10.0 nm with auto sensitivity and scan rate at 200 nm/min. Zero-order spectra were manipulated to generate first-order derivative spectra (D^1^) with a scaling factor of 100 and Δλ = 8. A calibration curve was constructed by plotting REM peak amplitudes at 428.3 nm versus its corresponding concentrations and the regression equation was calculated.

#### Application to laboratory-prepared mixtures

Synthetic mixtures of REM and AKDP were prepared by transferring different aliquots of their respective working solutions into 10-mL volumetric flasks, followed by dilution with distilled water. The mixtures were analyzed following the procedure under construction of calibration curve. The concentrations of REM were back calculated using the regression equation to determine how well the method detects REM in the presence of AKDP.

#### Application to Remdesivir IV

An aliqout of remdesivir IV solution was tansefered into a 50 mL volumetric flask, and the volume was completed to the mark with distilled water to obtain a stock solution with a nominal concentration of 100 µg/mL. Subsequently, appropriate dilutions of the stock solution were made to achieve concentrations within the linearity range. The REM concentration was calculated using the corresponding regression equation.

#### Application to spiked human plasma

Different aliquots of REM working solution were transferred into a series of centrifuge tubes. To each tube, 0.5 mL of plasma and 3 mL of acetonitrile were added. The tubes were vortexed for 1 min, followed by centrifugation for 30 min. The supernatant was collected and evaporated to dryness using a rotary evaporator. The residues were dissolved in an appropriate volume of distilled water, transferred to 10-mL volumetric flasks, and diluted to volume with water. A blank plasma sample was processed in the same manner.

## Results and discussion

Given the urgent need for a sensitive and selective method to quantify REM in intravenous formulations and plasma, both for monitoring its stability and for therapeutic drug monitoring (TDM) during COVID-19 treatment protocols, it was essential to develop a method capable of accurately identifying REM in the presence of its degradation products and/or metabolites. Studies have reported that REM is prone to hydrolysis, with the impurity profile of the pure drug and its finished product indicating the presence of two impurities that are potentially formed by hydrolysis with one of them a metabolite^3^. Therefore, before method development could proceed, it was necessary to prepare the degradation product and/or metabolite. Literature further corroborated REM’s susceptibility to hydrolysis, highlighting its greater vulnerability to alkaline hydrolysis compared to other degradation conditions^33,34^. Accordingly, stability investigations were conducted in an alkaline environment.

### Preparation of Alkaline-induced degradation product (AKDP)

The degradation of REM was carried out using NaOH and monitored through TLC with a mobile phase consisting of ethyl acetate, methanol, and ammonia (8:2:0.2, v/v/v). Complete degradation was confirmed by the appearance of a single spot after 2–3 h, so 2 h was the time used for complete degradation of REM.

The chemical structure of AKDP was examined using infrared (IR) and mass spectrometry (MS). As depicted in Fig. 1, REM contains two hydrolysis-prone functional groups: carboxylate ester and phosphamide. The IR spectrum of AKDP (Fig. S1b, Supplementary Material) displayed the disappearance of two characteristic peaks observed in the REM spectrum (Fig. S1a, Supplementary Material). These were the peaks at 1600–1700 cm^−1^ (C=O stretching) and 1200–1240 cm^−1^ (P=O stretching), suggesting cleavage of the phosphamide bond during hydrolysis. The MS analysis revealed a molecular ion peak at m/z 601 for REM (Fig. S2a, Supplementary Material) and m/z 290 for AKDP (Fig. S2b, Supplementary Material), corresponding to [M-1] in negative ion mode.

The combined IR and MS findings indicate that REM undergoes hydrolysis via phosphamide bond cleavage rather than carboxylate ester hydrolysis. The proposed hydrolysis pathway is presented in Fig. 1.

### Development and optimization of the spectrofluorimetric method

**Fig. 2:**
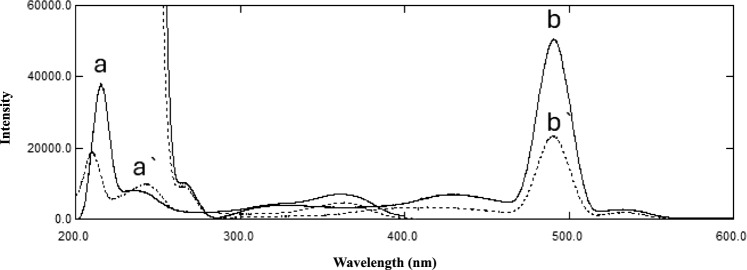
Excitation (a, a′) and emission (b, b′) fluorescence spectra of 50 ng/mL Remdesivir (––) and alkaline-induced degradation product (– – –) in water.

The excitation and emission spectra of 50 ng/mL of REM and AKDP solutions were recorded against water as a blank over the range of 200–600 nm. Various excitation wavelengths, including 232, 245, and 274 nm, were tested to achieve maximum fluorescence intensity for REM. Optimal sensitivity was observed at λ_ex_ = 245 nm (Fig. 2). Furthermre, the method parameters influencing fluorescence sensitivity were systematically optimized, including solvent, surfactant, sonication time, temperature, pH, scan speed, and bandwidth.

**Fig. 3:**
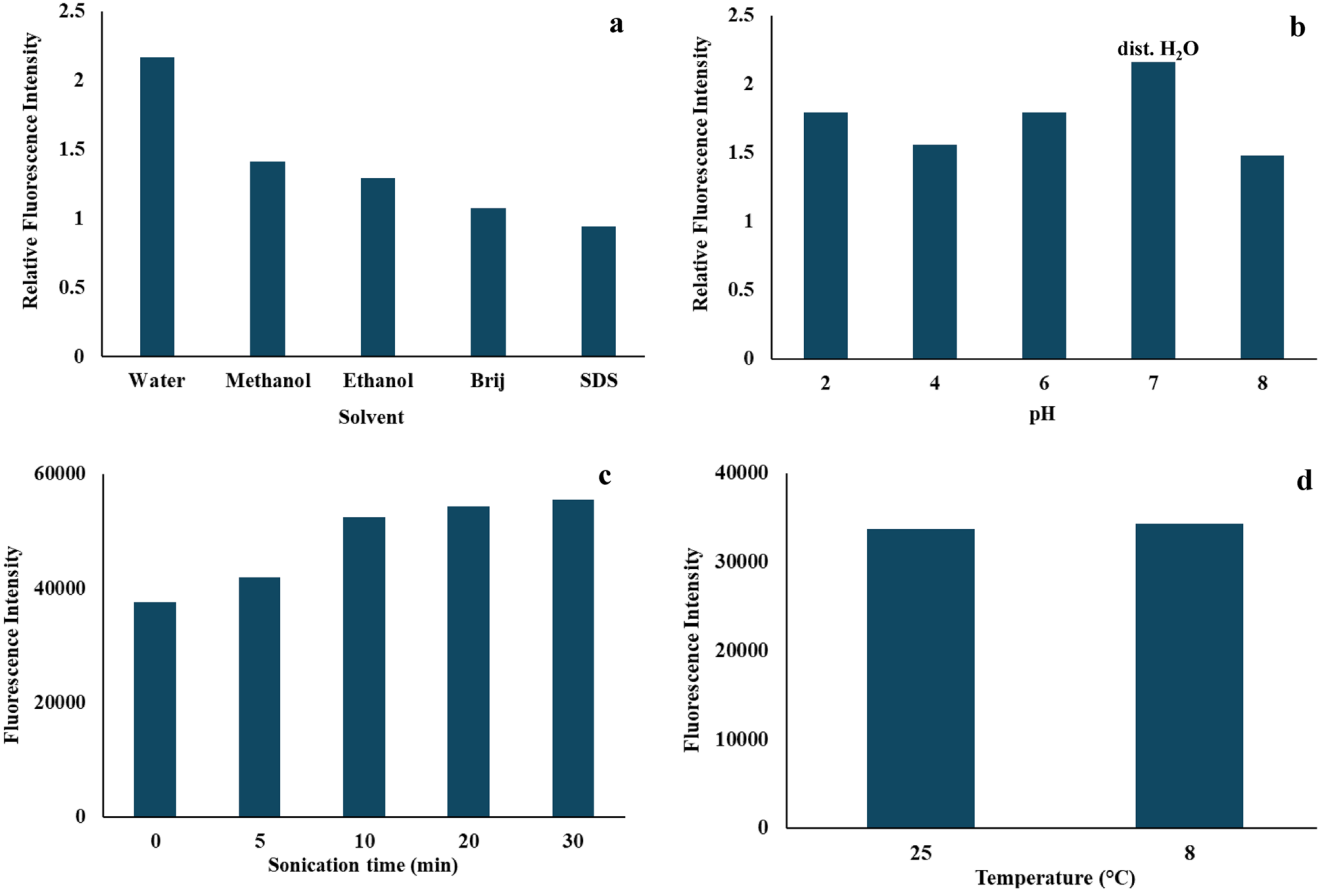
Optimization of Remdesivir (50 ng/mL) Fluorescence, (**a**) Solvent, (**b**) pH, (**c**) Sonication, and (**d**) Temperature.

Among the solvents tested (water, methanol, ethanol), water provided the highest fluorescence intensity, with no further improvement observed upon the addition of surfactants (SDS and Brij 35) (Fig. 3a). Buffer solutions at different pH levels (pH 2–8) were examined, but water remained superior in fluorescence intensity (Fig. 3b).

Sonication times ranging from 0 to 20 min were also investigated. Fluorescence intensity increased with sonication, reaching a plateau after 10 min; thus, 10 min was selected as the optimal duration (Fig. 3c). Temperature effects on fluorescence were minimal, with no significant differences observed between room temperature (25 °C) and refrigerated conditions (8 °C). For efficiency, room temperature (25 ± 2 °C) was chosen (Fig. 3d).

To achieve optimal spectral quality, various scan speeds (6000, 500, and 200 nm/min) and bandwidths (5 and 10 nm) were assessed. Higher scan speeds introduced noise into the spectra, while lower speeds produced smoother spectra. Conversely, reducing the bandwidth led to a decrease in fluorescence intensity. Based on these observations, the optimal parameters were determined to be a scan speed of 200 nm/min and a bandwidth of 10 nm, ensuring the best balance between signal clarity and intensity.

**Fig. 4:**
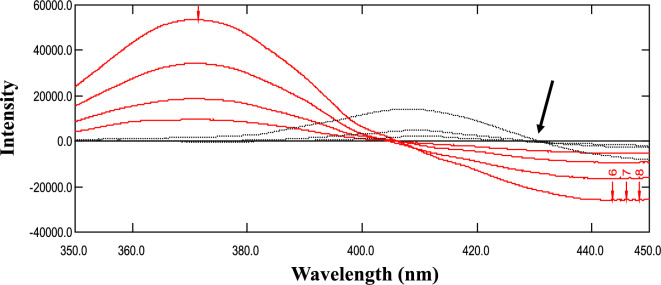
First derivative fluorescence spectra of Remdesivir (–––) and its alkaline-induced degradation product (.....) showing a zero-crossing point at 428.3 nm for Remdesivir determination.

The initial fluorescence spectra of REM and AKDP exhibited considerable overlap, with native λ_em_ at 407 nm and 432 nm, respectively, following excitation at 245 nm. To address this overlap, first-derivative spectra (D^1^) were generated. Different ∆λ values (4, 8, and 16) and scaling factors (10, 100, and 1000) were tested to optimize sensitivity and peak shape for REM. The optimal parameters were determined to be ∆λ = 8 and scaling factor of 100, which provided the best peak shape and sensitivity. Under these optimized fluorescence conditions, a zero-crossing point for the AKDP spectrum was identified at 428.3 nm (Fig. 4). This allowed for the determination of REM at 428.3 nm in laboratory-prepared mixtures containing varying proportions of REM and AKDP. The analysis results, summarized in Table 1, demonstrated a high recovery percentage (R%) for REM in the presence of up to 90% AKDP, confirming the method’s specificity.

**Table 1:** Determination of Remdesivir (REM) in the presence of its alkaline-induced degradation product (AKDP) using the proposed D^1^ spectrofluorimetric method in laboratory-prepared mixtures.

REM (ng/mL)	AKDP (ng/mL)	Recovery % of REM^a^
28.0	12.0	100.49
24.0	16.0	99.09
20.0	12.0	98.06
16 0.0	24.0	102.41
12.0	28.0	96.83
8.0	32.0	99.53
4.0	36.0	100.12
	**Mean ± SD**	99.50 ± 1.79

^a^Average of three determinations.

### Method validation

The developed method was validated in accordance with the ICH guideline Q2(R1)^38^, assessing parameters such as linearity, accuracy, precision, limit of detection (LOD), and limit of quantification (LOQ), as detailed in Table 2.

**Table 2:** Validation parameters data of the proposed D^1^ fluorimetric method for Remdesivir determination in the presence of its alkaline-induced degradation product.

Parameter	D^1^
Range (ng/mL)	3–120
Slope	233.66
Intercept	207.51
Regression coefficient (r)	0.9998
LOD (ng/mL)^a^	1.12
LOQ (ng/mL)^a^	3.67
Accuracy (mean R% ± SD)^b^	100.73 ± 1.51
Specificity (mean R% ± SD)^c^	99.50 ± 1.79
Repeatability^d^	0.84
Intermediate precision^e^	1.28
Robustness	
Temperature (25 ± 2 °C)^f^	0.86
Sonication time (10 ± 2 min)^f^	1.28
Stability (24 h)^g^	0.34
Spiked plasma (mean R% ± SD)^h^	97.64 ± 1.87

^a^Limits of detection “LOD” and quantitation “LOQ” are determined via calculations, LOD = 3.3*SD of residuals/slope, LOQ = 10*SD residuals/slope.

^b^Mean (n = 3) recovery % of five concentrations (25, 65, 80, 95, and 110 ng/mL).

^c^Mean (n = 3) recovery % of the laboratory-prepared mixtures.

^d^Intra-day precision, three concentrations (20, 70, and 100 ng/mL) measured three times within the same day.

^e^Inter-day precision, three concentrations (20, 70, and 100 ng/mL) measured three times in three successive days.

^f^RSD% for the fluorescence intensity at different conditions.

^g^RSD% for the fluorescence intensity at different times for 24 h.

^h^Average of three determinations.

The method demonstrated good linearity across the concentration range of 3–120 ng/mL (Figure S3, Supplementary Material). Accuracy was evaluated by calculating percentage recoveries for five concentrations (25, 65, 80, 95, and 110 ng/mL), each analyzed in triplicate. The results yielded a mean recovery of 99.97% ± 1.51, confirming the method’s accuracy (Table 2).

The study determined the repeatability (intra-day) and intermediate precision (inter-day) at three levels (20, 70, and 100 ng/mL) on the same day and three different days, respectively. Table 2 displays acceptable relative standard deviatin (RSD%) values that did not exceed 1.3%, proving good precision of the method. LOD and LOQ were calculated as 1.12 and 3.67 ng/mL, respectively, using the formulae 3.3σ/S and 10σ/S, respectively, where (σ) represents the residual standard deviation and (S) represents the slope of the calibration curve.

Robustness was evaluated to confirm the method’s resilience to small, deliberate variations. Minor changes in sonication time (10 ± 2 min) and room temperature (25 ± 2 °C) were introduced. Table 2 illustrates RSD% of intensity observed after modifying the factors, a maximum value of 1.28% confirmed the method robustness. Additionally, the short-term stability of REM standard solutions was examined over 24 h, demonstrating good stability.

### Application to pharmaceutical dosage forms and statistical analysis

Remdesivir in its IV formulation was determined using the developed D^1^ spectrofluorimetric method. The calculated R% of REM was statistically compared with values obtained from the reported method^33^. The results of the Student’s t-test and the F-test (Table 3) were not significantly different, indicating that the proposed method can be successfully applied to the analysis of REM dosage forms in QC laboratories.

**Table 3:** Statistical analysis of the proposed fluorimetric method and the reported HPLC method for the analysis of Remdesivir IV.

Parameters	Reported method^33^^a^	Fluorimetric method
Mean R% ^b^	99.06	98.99
Variance	0.681	0.727
n	3	3
Student’s t-test	–	0.098 (2.77)^c^
F-test ^c^	–	1.068 (19)^c^

^a^Agilent Zorbax Eclipse SB-C18 column, acetonitrile and distilled water (55:45, v/v, pH 4) as a mobile phase at a flow rate of 1.0 mL/min and detection at 240 nm.

^b^Average of three determinations.

^c^Tabulated t- and F- values (P = 0.05).

### Application to spiked human plasma

The method was further applied to determine REM in spiked human plasma. Various extraction procedures, including liquid–liquid extraction and protein precipitation, were evaluated for their effectiveness in recovering REM from plasma. Protein precipitation emerged as the most effective approach. Methanol and acetonitrile were tried as the precipitating solvent and acetonitrile provided superior recovery and sensitivity compared to methanol.

Using the optimized conditions, the developed method was successfully applied to analyze REM in spiked plasma samples, yielding satisfactory recovery results as presented in Table 2. These outcomes demonstrate that the method is reliable for quantifying REM in plasma and can be employed for its TDM during COVID-19 treatment protocols.

### Sustainability assessment and comparative evaluation of reported methods

Green Analytical Chemistry (GAC) represents a progressive discipline aimed at minimizing the environmental and health hazards associated with the use of conventional organic solvents in analytical procedures^39^. The application of GAC principles has expanded across multiple analytical platforms, including spectroscopic^40,41^, electrochemical^42^, and chromatographic techniques^43–45^. Among these, spectrofluorimetric methods are particularly advantageous for sustainable analysis, owing to their low operational costs, user-friendly nature, and significantly lower ecological footprint in comparison to chromatographic approaches^46,47^.

**Fig. 5:**
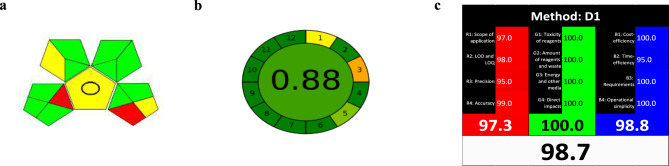
Greenness and whiteness assessments of the developed spectrofluorimetric method, (**a**) GAPI, (**b**) AGREE, and c) RBG12.

The environmental and sustainability aspects of the proposed spectrofluorimetric method were assessed using three comprehensive tools. The first was the Green Analytical Procedure Index (GAPI), which evaluates the environmental impact of each stage in the analytical process—from sample preparation to waste management—using a color-coded pentagram^48^. As illustrated in Fig. 5a, the GAPI assessment shows predominantly green and yellow zones, indicating strong environmental compatibility. Some red segments are visible, reflecting offline sample preparation and the absence of a waste treatment protocol.

The second tool used was the AGREE metric, which quantifies the method’s alignment with the 12 principles of GAC. The score is visualized in a circular diagram segmented into 12 parts, each shaded along a red-to-green spectrum depending on compliance^49^. The proposed method scored 0.88, as seen in Fig. 5b, indicating a high level of adherence to GAC principles.

The third evaluation involved the RGB 12 model, which integrates the principles of both green and white analytical chemistry (WAC). It groups 12 criteria into red (analytical performance), green (environmental impact), and blue (cost and practicality) domains^50^. The overall “whiteness” score for the method reached 98.7, as shown in Fig. 5c, confirming excellent sustainability across analytical, ecological, and operational dimensions.

In addition to its superior greenness profile, the proposed method was benchmarked against several previously published fluorimetric and HPLC methods for REM analysis (Table 4). The comparison considered technique type, solvent/reagent use, sensitivity range, application scope, and sustainability scores using GAPI, AGREE, and RGB12 tools.

**Table 4:** Comparison between the proposed Spectrofluorimetric method and the reported methods for determination of Remdesivir.

	Proposed method	Method^28^	Method^29^	Method^30^	Method^31^	Method^32^
Technique	Spectrofluorimetry	Spectrofluorimetry	Spectrofluorimetry	Spectrofluorimetry	HPLC	HPLC
Solvents/ Reagents	Water	Water	Ethanol	Water, and sodium dodecyl sulfate	Phosphate Buffer pH 3, acetonitrile, methanol, and water	Triethylamine, Water, orthophosphoric acid pH 3, and acetonitrile
Range	3–120 ng/mL	1–65 ng/mL	1–40 ng/mL	10–350 ng/mL	0.5–10.0 μg/mL	10–90 µg/mL
Application	Stability-indicating in dosage form and in spiked plasma	Dosage form and spiked plasma	Dosage form	Dosage form and spiked plasma	Stability-indicating in dosage form	Stability-indicating in dosage form
AGREE						
GAPI						
RBG 12						

The first three compared methods^28,29^, and^30^ employ spectrofluorimetry but do not investigate REM stability or detect its major metabolite (GS-441524). These methods are limited to quantifying REM in pharmaceutical dosage forms or spiked plasma without assessing degradation. In contrast, the proposed method includes a stability study under REM’s most labile conditions—alkaline hydrolysis—and successfully identify the degradation product (AKDP), which is also its active metabolite. This expands the method’s applicability to TDM and IV infusion quality control.

In terms of sensitivity, the proposed method shows comparable or superior performance, covering a wide linear range (3–120 ng/mL) suitable for plasma-level quantification. Notably, it uses water as the solvent, which is the greenest choice available, unlike ethanol (used in Method^29^) or SDS surfactant (used in Method^30^). These factors are reflected in the enhanced RGB12 and GAPI scores achieved by the proposed method, as seen in Table 4.

While HPLC-based methods (Methods^31^ and^32^) are considered stability-indicating, they did not characterize the degradation product. Moreover, they are not applicable for plasma quantification due to their lower sensitivity (μg/mL range), which limits their utility for TDM. Additionally, the use of acetonitrile, methanol, and triethylamine in their mobile phases significantly reduces their environmental friendliness, as indicated by their lower AGREE and RGB12 scores.

The developed spectrofluorimetric method successfully combines the key strengths of both method categories—matching or exceeding the sensitivity and greenness of fluorimetric methods while also serving as a stability-indicating method akin to HPLC. Unlike previously reported methods, it provides structural characterization of the alkaline degradation product (AKDP) using IR and MS, confirming its identity as REM’s active metabolite (GS-441524). The method enables the selective determination of REM at the nanoscale level in pharmaceutical formulations and human plasma, making it suitable for both TDM in COVID-19 patients and QC of IV infusions.

## Conclusion

A robust, highly sensitive, and environmentally sustainable spectrofluorimetric method was successfully developed and validated for the quantification of REM in IV formulations and spiked human plasma. The method uniquely incorporates the detection of REM in the presence of its alkaline-induced degradation product and active metabolite (GS-441524), whose structure was confirmed through IR and mass spectrometric analyses. Compared to previously reported fluorimetric methods, the proposed technique offers superior applicability as a stability-indicating assay, resolving spectral overlap and enabling accurate quantification of REM even under degradation conditions. Unlike reported HPLC methods, it also characterizes the degradant structure and demonstrates sufficient sensitivity for TDM at nanogram levels—capabilities not achieved in earlier approaches. This method demonstrated high specificity for REM quantification in solutions and human plasma even in the presence of its degradation product/metabolite, making it an effective tool for QC and TDM. The method outperforms published techniques in terms of greenness, as evidenced by favorable scores across GAPI, AGREE, and RGB12 assessments, and benefits from using water as a solvent. Its simplicity, cost-effectiveness, and compliance with green analytical chemistry principles make it a highly practical and eco-conscious solution for both QC of IV infusions and clinical monitoring of REM, supporting safe and effective treatment protocols for COVID-19.

## Supplementary Information

Supplementary Information.

## Data Availability

All data generated or analysed during this study are included in this published article and its supplementary material files.
