# Supplementary material for: Eco-friendly spectrofluorimetric determination of remdesivir in the presence of its metabolite in human plasma for therapeutic monitoring in COVID-19 patients
Source: Sci Rep. 2025 Jun 23;15:20251. doi: 10.1038/s41598-025-05198-4 (PMC12185737; doi:10.1038/s41598-025-05198-4)
Supplement: Supplementary file 1 — Supplementary Information. [file 41598_2025_5198_MOESM1_ESM.pdf]

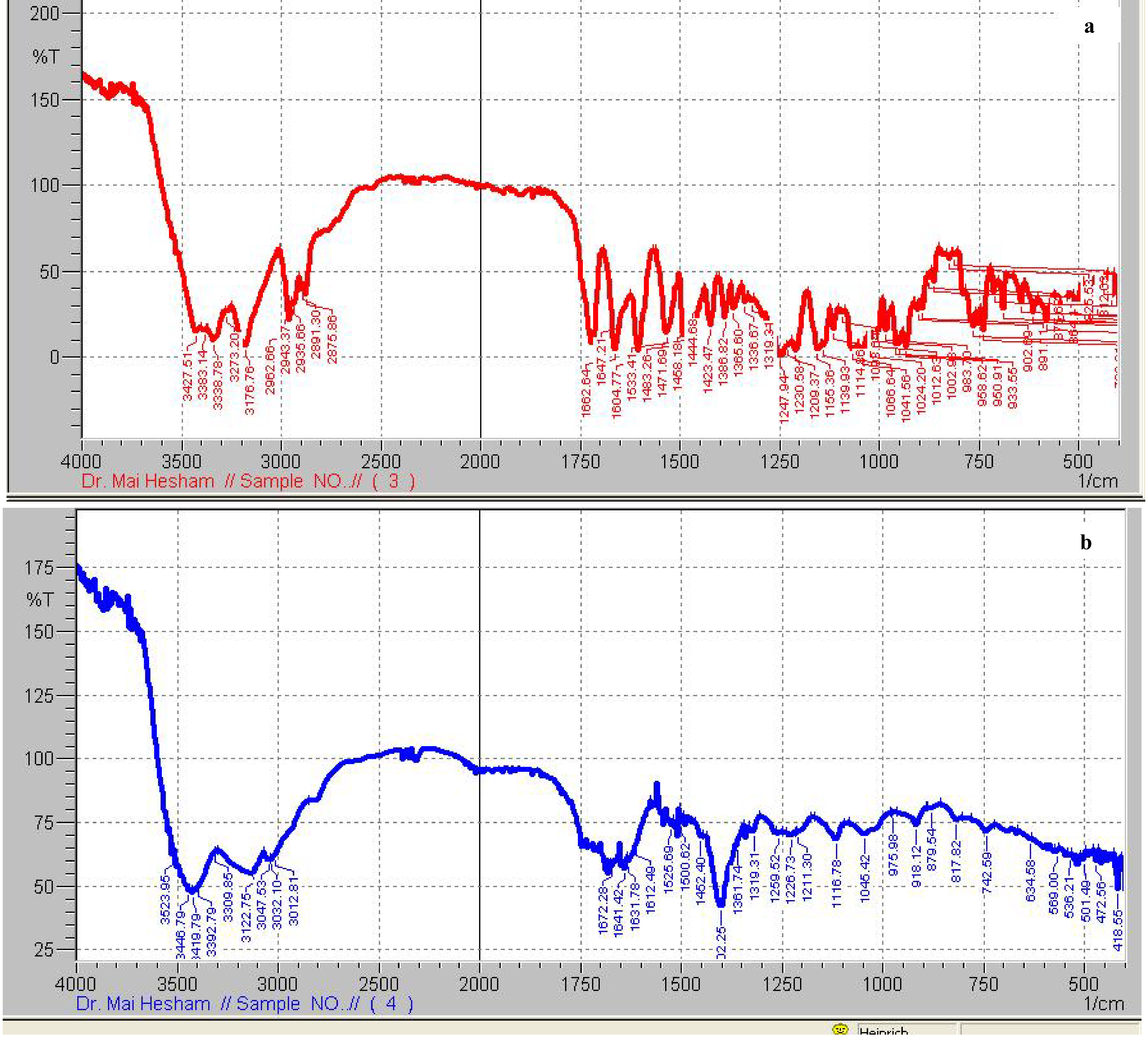

Figure S1. IR spectra of (a) Remdesivir and (b) alkaline-induced degradation product.

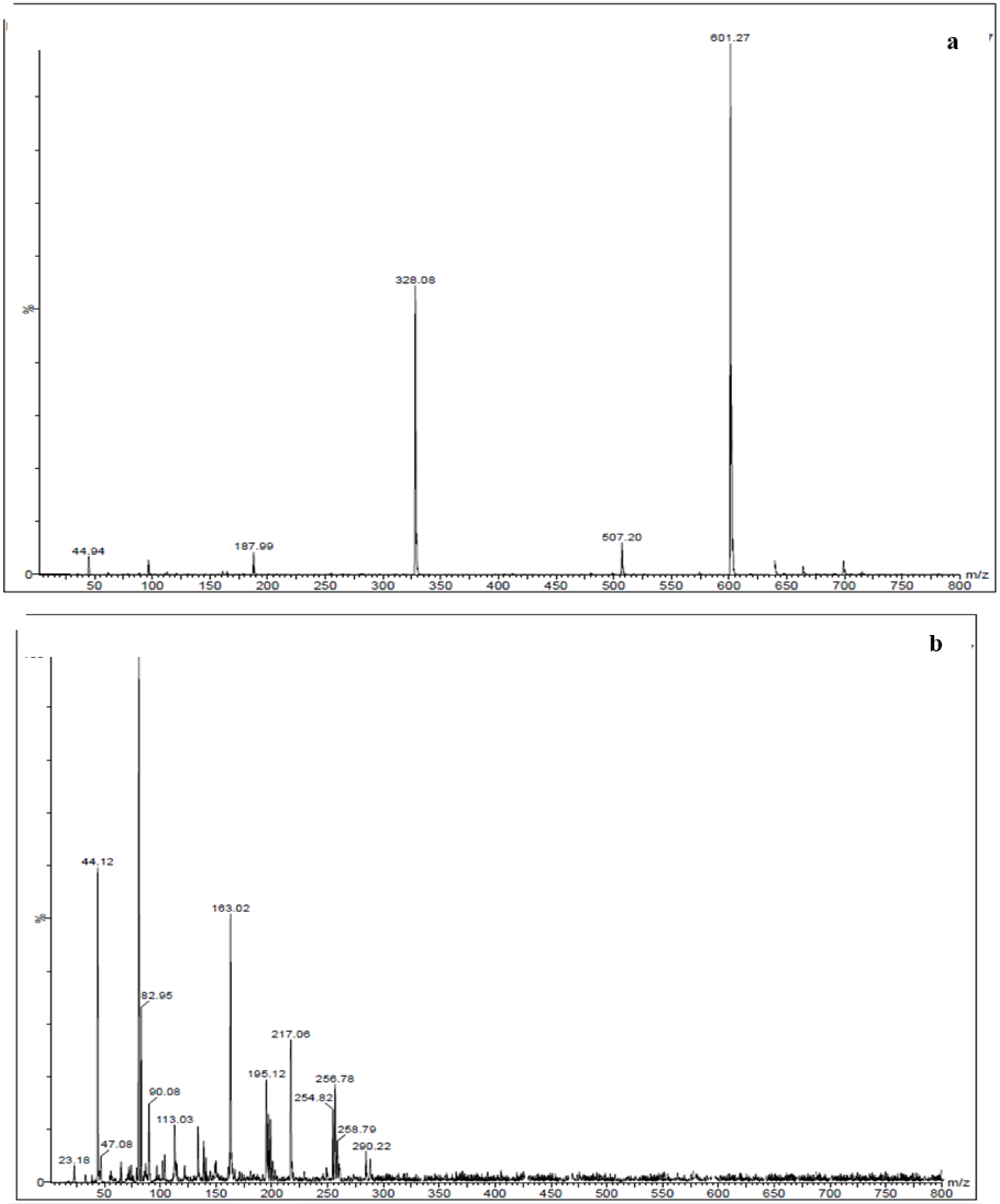

Figure S2. Mass spectrum of (a) Remdesivir and (b) alkaline-induced degradation product.

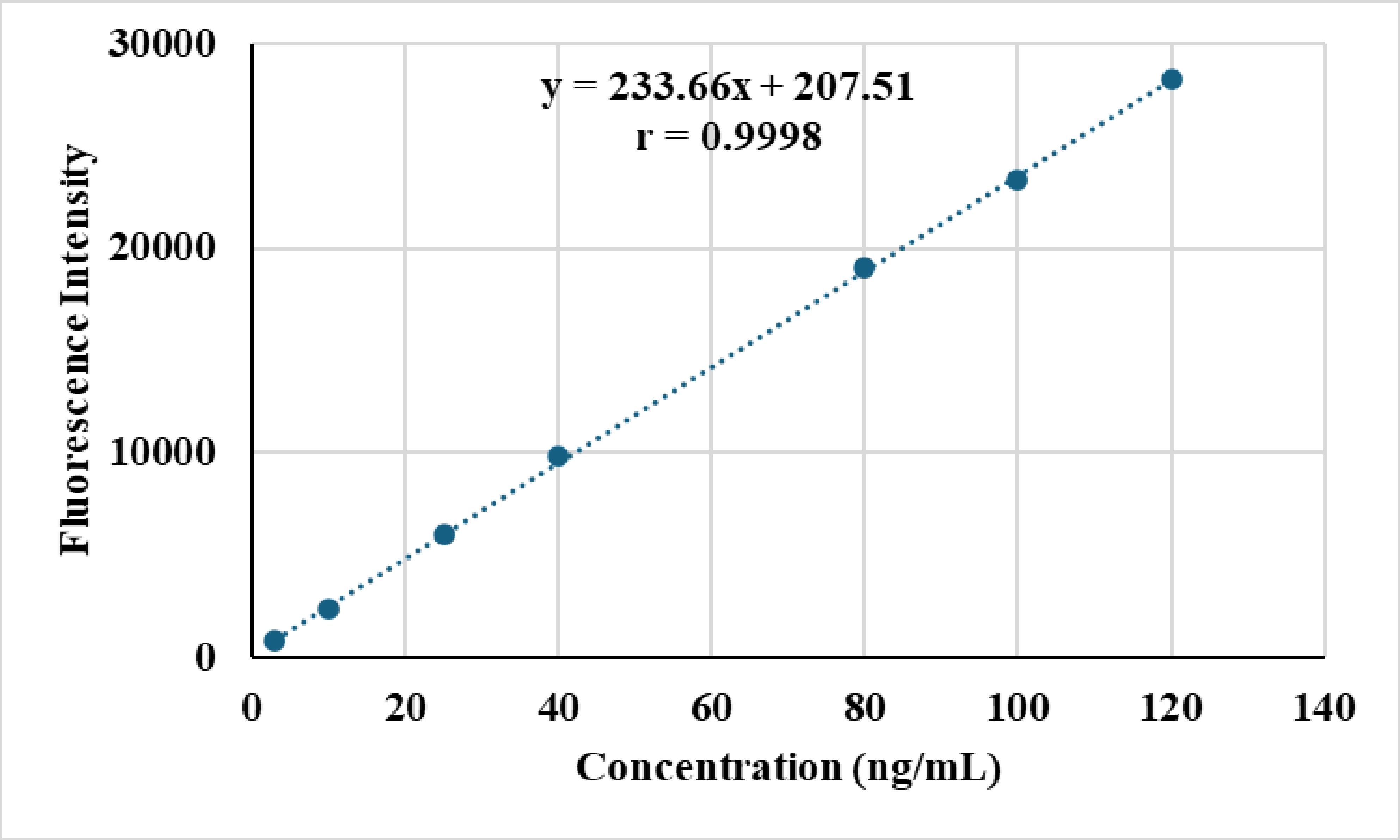

Figure S3. Calibration curve of fluorescence intensity versus REM concentration (ng/mL)
